# Glanders and Farcy in the Horse

**Published:** 1846-07

**Authors:** 


### ART. III.

*Glanders and Farcy in the Horse.* By WILLIAM PERCIVAL, M.R.C.S., Veterinary Surgeon of the 1st Life Guards, &c.—*London,* 1845. 8vo.

THE diseases of those domestic animals which are the constant associates of man are entitled to our best consideration. Comparative anatomy has contributed largely towards the advancement of human physiology, in dispelling error and eliciting truth in various points connected with the economy of the functions; so likewise comparative pathology may be made subservient to that of the human subject. Until recently, however, veterinary medicine has received but little aid from those whose province it was to cultivate it; for the researches of all the older writers upon this subject are a singular compound of fanciful speculation and irrational empiricism. We seek in vain in veterinary works for unity or precision in the various accounts of diseases of the lower animals; in which the pathological conditions are either wholly overlooked, or else so imperfectly described that we turn away in despair from descriptions which seem to have no other purpose than that of mystifying the nature of disease.

Brute pathology is still almost wholly unexplored; yet there is no department of science which offers a wider field of interest and instruction to the medical philosopher than the pathological history of the mortal diseases of those animals which approach nearest in the scale to man. The extreme facility with which several of those maladies are transmitted from the brute to the human subject, and their invariably fatal termination in the latter, are facts sufficient to enlist our sympathy and arrest our attention. If further stimulus were wanting, we have it in the consideration that a rigid inquiry into the causation of the class of diseases referred to cannot fail to expand our views by disclosing new facts, elucidating points hitherto obscure in human pathology, and otherwise contributing to improve the science of medicine. In the most important of all the branches of medicine, therapeutics, a virgin field of the greatest possible value lies here before us; where at least we may, with a safe conscience, and without trepidation, endeavour to ascertain the natural history of diseases, the remedial powers of nature, and the positive effects of medicines. Although we shall not be justified in reasoning positively from the results thus obtained, as to results to be obtained in the human organism, it cannot be doubted that the strong analogical grounds thence derived will far exceed the majority of those on which we are accustomed to rely in practical therapeutics.

The murrain, an epidemic disease of cattle as fatal and as mysterious in its operations as the cholera, is transmissible to man. Yet this malady has not been described in medical works, and doubtless is considered as foreign to the subject of medicine as glanders was some twenty years ago. Men have died of this disease; and cases are recorded in which animals and their attendants have been attacked with the epidemic, exhibiting symptoms precisely similar, under which both have finally succumbed. "Those persons," says Dr. Slare, in an account of this epidemic, published in the thirteenth volume of the 'Transactions of the Royal Society,' "that carelessly managed their cattle, without due regard to their own health, were themselves infected, and died away like their beasts."

It were easy to relate facts showing the great importance of the subject of brute pathology with reference to state policy, as well as in a medical point of view; but as a branch of hygiene its importance is so obvious that we feel confident the day is not distant when a "Board of Health" will see the necessity of preventing, by stringent measures, so enormous and palpable an evil as that of animals infected with a mortal disease, and snorting a poisonous virus from their nostrils, from plying in the crowded thoroughfares of the metropolis and of other large towns.

Taking a lively interest, as we do, in the progress of comparative pathology, we looked forward with no small degree of pleasure to the perusal of Mr. Percival's contribution to that science now before us. A monograph on glanders and farcy emanating from such a respectable source led us to expect more, perhaps, than the present condition of veterinary medicine warranted. Be that as it may, however, the work before us has not fulfilled our expectations. It may be useful to refer to as an epitome of what has been written on these diseases in the quadrumana; but beyond this its utility does not extend. Of novelty it contains nothing. It leaves the pathology of glanders precisely where it was before; and, in fact, fulfils no other end than the subordinate one of supplying a text-book of what has been already done upon the subject. As, however, the volume may be considered to express the views of the veterinary profession on the nature of glanders in the *horse,* a summary of the principal facts it contains may be read with profit, and will tend to facilitate the study of the disease in man.

Mr. Percival's work consists of three parts. The first embraces an account of the "symptoms, diagnosis, varieties, causes, seat, and nature of glanders." In the second are described the "symptoms, causes, seat, and nature of farcy;" and the third is occupied with "the treatment of glanders and farcy." In the body of the work there are about twenty pages describing the author's own views on the subject, which are, in point of fact, but an amplification of those of M. Leblanc, of Alfort.

The following is Mr. Percival's definition of glanders: "Glanders consists in a discharge from one or both nostrils of matter which, by transfer or inoculation, will produce the disease in another animal (of the equine or human species), and which discharge is sooner or later accompanied by vascular injection and chancrous ulceration of the Schneiderian membrane, by tumefaction of the submaxillary lymphatic glands, and by farcy." This definition is imperfect. All mention of propagation of the disease by *infection*, or through the medium of the atmosphere, is omitted; and to prove that the disease is not altogether confined to the "equine or human species," we may mention the fact, which came under our own observation, of a dog which sickened and died with symptoms very similar to those of glanders, after inoculation with the virus of that disease taken from the horse. We may also refer to the 'Bull. de l'Acad. Roy. de Méd.,' (31 Dec., 1839, et 15 Fevr., 1840,) in which M. Renault relates two cases of glanders produced in the sheep by inoculation; and to M. Hamont's 'Memoirs on Glanders,' where the disease is described as having been transmitted, by inoculation, to a lion and to dogs.

Symptoms. The symptoms of glanders in the horse present several peculiarities which are not met with during the progress of that disease in the human subject. One of the most singular of these is the apparent restoration to health of the animal after the febrile indisposition usually present during the incubation of the disease, has subsided, and the discharge from the nostrils is freely established. This peculiarity is the cause of much mischief; for it blinds the incautious and unprofessional observer, who naturally enough supposes there is nothing the matter with the horse further than "a running from the nose and kernels," which may be the result of cold; and hence the propagation of the disease to other animals is facilitated. How different is the progress of glanders in man! Here its brief course is unbroken by any of these deceitful signs of returning health; and the only termination of his sufferings which the patient can hope for is death itself. As a general rule the first external physical sign of the disease developed in the quadrumana is the enlargement of the submaxillary lymphatic glands; but unfortunately the first symptom that *attracts notice* is the discharge from the nose. This discharge is at first scanty and limpid, and usually trickling from one nostril only. In the course of a day or two it becomes thickened with mucous and albuminous matter, loses its watery appearance in a great measure, and quickly passes from a limpid to a glairy condition, finally to become of a yellow tint,—and this terminates the first stage. The second stage commences with decided appearances of purulent matter, scanty at first, but soon flowing abundantly; this gradually thickens, becomes as glutinous as birdlime, impedes respiration, and produces a snuffling noise in the nostril not unlike the mucous or bronchial rhonchus. This latter symptom is decisive as to the nature of the disease. As the destructive ulceration of the bones and cartilages of the nose advances, the discharge changes from yellow to green, or to a dirty brown or leaden colour, streaked with blood, and gives rise to a horribly fetid and disgusting smell, which marks the last stage of the complaint, and continues unabated until dissolution takes place. This is the regular course of the acute form of glanders: when it assumes a chronic character the discharge may continue from the first aqueo-mucous, or turn to a nasal gleet only. The discharge of the acute form when analysed shows albumen in abundance, from the presence of which, perhaps, it derives its glutinous character. The diseased parts present the usual appearance of inflammation—vascular injection, thickening of the pituitary membrane, infiltration of the cellular tissue, ulceration, &c. Of these different lesions, the only one which has a specific character is the ulcerative process, and that presents one or two striking peculiarities pathognomonic of the disease. We shall relate these in the words of the author:

"Introduce into a scratch the *virus* taken from a glandered animal, and the result will be that, losing all disposition to heal, the lesion will inflame and secrete an ichorous matter, and become converted into a transparent vesicle, surrounded by an *areola* or circular blush upon the membrane. The next day the vesicle has broken, and we perceive in the place of it a pale, foul, superficial ulceration, which in the course of another day acquires the genuine character of the glanderous *chancre*, an elevated, circular, pinkish border, including a base of faint yellow albuminous matter, which on being wiped or irritated bleeds, and on the matter being removed exposes, when the ulcer is deep, the bare cartilage beneath; when superficial, a red, spotted, rugged, foul, bleeding bottom. From its tendency to spread, the ulcer speedily loses its circular figure, for one too irregular and variable to admit of precise description; it has, in fact, now become a foul, spreading ulceration, extending on every side, coalescing with similar ulcerations in its vicinity, having for its base the cartilage of the *septum nasi*, which alone, from its comparative insusceptibility of the ulcerative action, puts a temporary arrest to its devouring activity."

In virulent forms of the disease the turbinated bones are often ulcerated through into the nasal sinus, and Mr. Percival has seen cases in which these bones have been almost entirely removed, through the ravages of the ulcerative and exfoliating processes. The chronic form of glanders presents a different variety of ulceration but equally characteristic, which is called by veterinary writers "miliary ulceration," giving to the diseased parts the appearance of worm-eaten wood, or as if the membrane was covered with pin holes. This condition is the result of the degeneration of miliary tubercles, according to M. Dupuy. He describes the ulcerations as being superficial, having thin, irregular edges, and following the course of the large veins upon the septum; they are frequently mistaken for the dilated orifices of the mucous follicles. Tumefaction of the ala nasi is a frequent symptom of the acute form of glanders, and when present is highly characteristic of the disease. It is seldom seen in the subacute or chronic forms. The left or near side of the head is considered by several veterinary writers, and amongst others by M. Dupuy, to be much more frequently the seat of the disease than the right; an opinion which is not substantiated by facts, and which our author very properly discards, in the absence of more precise data than the mere assertions of writers however respectable.

Diagnosis. The diseases with which glanders is most likely to be confounded are catarrh, nasal gleet, and strangles; but the characteristic features of the former when developed are so striking that a mistake in the diagnosis is not likely to occur to any careful observer. "The signs by which the disease may be known," says Solleysel, "are when a horse too old to be troubled with strangles, without a cough, voids matter by the nose, and has a kernel sticking to the bone; and besides in glanders the matter usually flows from one nostril, whereas in a cold it runs almost always out of both." It sometimes happens, however, that some local disease about the head, neck, or lungs, excites a discharge from the nose, and when this discharge flows from one nostril only, it is likely to be mistaken for glanders. A carious tooth, for example, may produce symptoms of this kind; hence the necessity of carefully examining into the history and origin of these symptoms before forming the diagnosis, and in doubtful cases time should be allowed for their development before the animal is condemned. The most effectual way, however, of establishing a diagnosis is by inoculation of another animal, in good health, with the matter taken from the suspected glandered sore. The result of this experiment will finally settle the question.

Varieties. Mr. Percival recognizes three varieties of glanders—the *acute, subacute,* and *chronic*—a division which has the merit of conciseness, and is preferable to the involved and complicated classification of the early veterinary writers; for, after all, the disease is essentially the same in all its types, and its variations are merely in degree, and are modified according to the tissue and locality in which it is principally seated, as the lungs, the schneiderian membrane, the sinuses, &c. Mr. Percival considers what he calls "inoculated glanders" to be the best example of the acute form of the disease, the symptoms of which we have already described; the "typhoid glanders" of French writers being no other than a malignant form of this variety.

Mr. Percival describes a variety under the name of "*pulmonary glanders,*" which supervenes on the subacute, and even on the chronic species, whenever the lungs, in which the disease has been either unceasingly or in relapses creeping on all the while, have arrived at a point of disorganization to fail in their functions, and so as to create constitutional irritation. The author has remarked in these cases that the disease has generally confined itself to the lung of the side corresponding to the affected nostril. The animal's health and strength continue to decline for months, and sometimes for years before the final break-up takes place. In this variety death is never occasioned by suffocation as in the acnte form, but, to use the author's expression, "through consumption of the lungs by the disease, assisted by a wearing, hectic sort of fever, the same as is seen in human phthisis pulmonalis; and, moreover, bearing a resemblance to that mode of ending life, inasmuch as the brute as well as the man retains his senses to the last."

We wish the author was more precise in explaining what he means by "consumption of the lungs." Does he mean tuberbular phthisis, or merely pulmonary abscess? If the latter, which we suspect to have been the cause of death in these cases, there was no necessity for a separate description of "pulmonary glanders," which is nothing else but an extension of the disease from the nostrils to the lungs in cases of long standing, and does not require to be described apart any more than "glanders of the frontal sinus," or "glanders of the schneiderian membrane."

*Subacute glanders* is the variety most frequently met with. After the disease is established the symptoms gradually subside, and remain in a state of inactivity for a considerable period, until some occurrence takes place to derange the health and excite the disease into renewed action. It then assumes the acute form, and progresses rapidly to a fatal termination.

*Chronic glanders* differs both symptomatically and pathologically from the acute and subacute forms of that disease. As regards symptoms, it differs from them in the absence of anything like inflammation, or vascular injection, or chancre, or, in fact, of any perceptible change in the aspect of the schneiderian membrane denoting morbid activity. The flux from the nose and the submaxillary tumefaction being the only indications of a deviation from health. It differs pathologically from the varieties above named in selecting for its especial seat the lining membrane of the sinuses of the head. Some veterinarians allege that a slight prominence is to be felt over the frontal sinus, and when the part is tapped with the knuckle the sound elicited is duller than natural, and the parts tender to the touch; but this appears to be mere speculation, and is not supported by facts. It is in this variety of glanders that the peculiar ulcerative process we have noticed in a former page, called "miliary ulceration," is observed.

During the perusal of this section of Mr. Percival's monograph we have been forcibly struck with the two leading faults which prevail throughout the work, and which materially detract from its merits. The faults we allude to are want of order, and a vague, loose method of expression in his pathological descriptions. The *lucidus ordo* seems to be unappreciated or not understood by the author, and, as might be expected, confusion and frequent repetition are the result. Although there are separate sections devoted to descriptions of the "symptoms" and "diagnosis" of glanders, in the present section—which, without inflicting any injury on the history of the disease might have been incorporated with the first,—we have the same story over again. We have detailed accounts of symptoms already described, and as a wind-up to the section we are favoured with two pages on diagnosis, although that subject had been already discussed in the preceding chapter.

Causes. The origin of glanders is a subject of extreme interest, and seems, from the very dawn of veterinary pathology, to have been a *questio vexata* with the writers on and professors of the veterinary art. Although the contagious nature of the disease is now established beyond all doubt, there are still writers, and respectable practitioners too, who zealously support the opposite opinion. The arguments of this class of reasoners are frequently put forth with such total disregard to the commonest rules of logic as well as of physiology, that the dispassionate reader is inclined to view them rather in the light of party contentions than as attempts at the solution of an important question in science.

Mr. Percival considers the causes of glanders to be twofold—predisposing and exciting. Horses of an inferior breed, ill-fed, ill-conditioned, and over-worked, are highly obnoxious to this disease; the ass more so than the mule—the mule than the horse; and it appears from statistical tables that horses of the adult or middle ages are much more frequently attacked than foals and geldings. Mr. Percival does not seem to be acquainted with the valuable memoirs of M. Hamont, read before the Academy of Sciences in 1842, nor of the interesting work of Dr. Levin on the transmissibility of glanders from animals to man, reviewed in this Journal (January, 1842). Mr. Percival says the disease "is absolutely unknown in hot climates, in Arabia and Africa, to which we may add India," whereas M. Hamont states the very reverse; and from his position as director of the veterinary school at Abon Zabel, in Egypt, ought to have some weight. The latter writer describes glanders as of frequent occurrence amongst the inferior horses of the poorer Bedouins, whereas the blood horse in Egypt scarcely ever falls a victim to the disease. He considers glanders to be a disease of privation (*misère*), and only attacks impoverished animals, whose constitutions are broken down by over-work and bad feeding, or those of a deteriorated breed. Solleysel, who wrote in 1669, seems to have had a pretty shrewd notion of this disease. He describes glanders as one of "the most contagious distempers to which horses are obnoxious; for not only does it communicate its venom at a small distance, but infects the very air, and seizes on all horses that are under the same roof with him that languishes from it. There are several kinds of glanders, some of which are not so infectious as others, though there are none that ought not to be suspected." If the foregoing statement was qualified by the remark that all horses are not equally susceptible of the disease, and that instead of there being several kinds of glanders (for it is essentially the same disease in all its forms), it is more contagious at one period than at another, and that its transmission from animal to animal is influenced by the ordinary laws of animal poisons, it would not be far from the truth.

We cannot give a better illustration of the vague and indefinite expressions and want of precision of Mr. Percival's style, than by noticing the indiscriminate use by him of the words "contagion" and "infection." In the same work they are sometimes used synonymously; sometimes to express different meanings; so that it is almost impossible to arrive at what the author really means. The term "contagion" is usually meant to imply the propagation of disease by actual contact, in contradistinction to "infection," which is understood to express the propagation of disease through the medium of the atmosphere—at least this gives a distinct and definite meaning to each of these terms, and would render the author's views much more intelligible. In the following remarks we shall use them in this signification.

Amongst the non-contagionists the most formidable were Dupuy and Coleman, and certainly these names are a host in themselves. Coleman says that not one horse in ten thousand receives the disease through contagion! He adds "the poison of glanders is bred and diffused in an atmosphere rendered impure by repeated respiration, and by gaseous impregnations from the dung, urine, and perspiration emitted in hot and foul stables." In support of this view he alleges that it rarely or never spreads amongst horses at pasture, though a glandered subject may have been grazing amongst them. With every respect for Mr. Coleman's name we dispute the accuracy of the above statement, and cannot but believe that if he had written at a later period he would have materially modified his views. When we find such passages as the following in his work, totally at variance with *facts,* it is not too much to suppose that further experience would have led him to alter his opinion, that not one horse in ten thousand died of glanders through contagion. "*If,*" says Mr. Coleman, "*man had been susceptible of contracting diseases from horses,* oxen, hogs, sheep," &c. &c.! We believe, however, that Mr. Coleman did subsequently admit the transmissibility of the disease from the horse to man. As a specimen of the kind of arguments often advanced by the non-contagionists we submit the following from a work on glanders, by Mr. Smith, a veterinary writer of some note: "If the mucus issuing from the nostrils of the horse be so infectious as it is generally supposed, how is it that those animals which have access to the places where they stand, and in which they are frequently confined, escape the disease—especially dogs, who also feed on such horses immediately after death? It is very well known that horses are affected with hydrophobia when bitten by a mad dog. It is reasonable, therefore, to suppose that if glanders were equally contagious the disease would be equally reciprocal." This writer evidently forgot, or did not know, the recognised fact that all animal poisons are governed in their operation by certain laws peculiar to themselves; that one animal is more susceptible than another; and that the same animal is more susceptible at one time than another. We may inoculate the disease without success from insusceptibility of the subject inoculated, as we see every day in vaccinating children, on the same principle that we may administer the same quantity of aloes to two horses, and effect violent purgation in one, but make no impression upon the other. The fact that the most virulent animal poison may be swallowed with impunity, whereas if applied to an abraded surface would produce instant death, seems also to have been unknown to this writer, although familiar enough to most people. M. Leblanc, of Alfort, an eminent veterinarian of the French school, believes that glanders and farcy are infectious in all their forms, though in different degrees.

Mr. Percival, who was educated in the Coleman school, is now a decided infectionist, although in his early life he was opposed to that view. A series of facts illustrative of the transmission of glanders through the medium of the atmosphere, occurring under his immediate observation, convinced him of the fallacy of his former views, and led him to adopt those he now entertains.

The 1st Life Guards, to which the author is veterinary surgeon, was ordered on the 14th March, 1833, from the Regent's-park Barracks to out-quarters at Barnet, Highgate, and Hornsey. The horses were in good health at the time, and no case of glanders had occurred in the regiment since the author joined, about seven years previously. They remained in out-quarters eight days, and the day after their return the colonel's horse first, and subsequently three others, became infected with glanders. It appears that these places are notorious for propagating glanders; which is accounted for by the number of hard-worked horses crowded together in them constantly, to supply the public conveyances on the great northern roads. On the horses returning to their own stables, and those diseased being removed from the healthy animals, the malady soon disappeared.

Mr. Percival's view of the *modus operandi* of contagion (infection?) in this as in all other similar instances is as follows: "Glandered horses having formerly tenanted these stables, the animals infected became so by inhaling the effluvia caused by moisture and heat to arise from the dessicated besmearments, and that these effluviæ entering with the air into the animal's air passages, therein became absorbed, and thus infected his system; the contamination, therefore, mediate instead of immediate, breaking out afterwards in the form of glanders and farcy." The author does not believe that infection was produced by contact of the animal with the debris of glandered matter adherent to the woodwork of the stable or elsewhere. He believes that the aerial membrane is the medium through which the virus of glanders becomes introduced in cases of infection; in fact, that it is inspired. Here again we have to complain of our author's vicious style. From a carelessness of expression he is made to contradict himself in the most direct manner. After stating as we have just seen that it was by inhaling the morbid effluvia that glanders was developed in the cases above mentioned, he begins the following paragraph with the query—"Can glanders be propagated through the medium of the air?" and answers it thus: "I think myself it is possible, *though a very unlikely incident*, that the air may become the medium of contagion!" We should like the author to explain what he means by "effluvia" if it is not "air" modified, but still air. However, on reading a little further, we discover that the author was in a reverie, from which he awakes with this notable reminiscence: "Indeed, if we come to consider, the air ought to be regarded as the communicating medium in the cases of stables, &c.!" and a few lines further on, when the author's memory had more completely returned, he says, "the probability, therefore, is that the air plays a more important part in the ordinary work of contagion (infection?) than we are in the habit of imagining." (p. 238.) Not a bad *amende* this for the previous obliviousness. This, however, is not all. At page 243 we find the author making further admissions as to atmospheric infection, which we cannot resist noticing, especially as they favour our own views of one of the causes of glanders. Coleman, as the reader will recollect, believed that the ordinary source of glanders was "a poison generated in a confined atmosphere, out of exhalations from the breath, the dung, the urine, and the perspiration of horses pent up in it;" and this is what Mr. Percival calls the "miasm of the stable." In commenting upon Coleman's view, the author observes:

"To my mind, however, Coleman's own reasoning on the *modus infectandi* of this poison is in every way sufficient to prove that the disease once generated is capable of spreading by contagion, and through the medium of the atmosphere, too, from one horse to another. If the atmosphere of the stable, charged as we know it to be with humidity, can carry a miasm from the excretions and secretions into the nose of the horse, sufficiently concentrated to produce glanders, is there any good reason why the same atmosphere may not convey the virus of glanders itself emanating from the nose or lungs of a glandered horse? Surely that which can conduct poison from the dung or urine upon the floor of the stable can transport virus from one horse's nostrils into those of another."

While rejecting the doctrine of Coleman, the author has at length arrived at a correct view of the case; and in spite of this contradiction we are willing to give him credit for the correctness of these incidental observations.

We have ourselves paid considerable attention to the etiology of glanders for several years past, and we shall now state in a few words the result of our observations as to the origin of that disease. It appears to us that glanders may be produced in three different ways: 1st, Spontaneously, through privation, over-work, and foul stabling; 2d, by infection, i. e. by inhalation of the effluvia of a glandered animal, or of the atmosphere of a stable wherein a glandered animal was kept; 3d, by contact or inoculation.

1. We have observed that the same depressing agents which predispose to fevers of a typhoid character in man, by lowering the *vis vitæ*, predispose to glanders in the quadrumana, especially when broken down by age, over-fatigue, bad feeding, and more particularly if the animals are of an inferior breed. We have seen the disease on several occasions developed under these circumstances in large, airy, and well-ventilated stables, and perfectly coincide with the statement of M. Hamont that moisture and cold, narrow, and ill-ventilated stables will not of themselves produce glanders. Take a horse of a good breed and in health, feed him well, give him ordinary exercise, and put him into the coldest and dampest stable you can find, provided it is not glandered, and we feel confident he will not become diseased; but if you over-work an inferior horse, feed him badly, and keep him in the best ventilated stables that Mr. Coleman could devise, the animal will in all probability become glandered; of course allowing for the caprice of susceptibility. We have also noticed that the higher breeds of horses rarely ever generate the disease, i. e. spontaneously, unless very old and then over-wrought. We perfectly agree with M. Hamont that the *original* causes of glanders do not exist in stables, and that the habitation exerts but a secondary influence towards its development.

2. That glanders is transmissible from one animal to another through the medium of the atmosphere we firmly believe, and we have adopted this view from experience, for originally we did not entertain such an opinion. There is now no doubt in our minds that glanders is infectious, and can be transmitted from animal to animal through the inhalation of a contaminated atmosphere, precisely as hooping-cough or scarlatina passes from one human subject to another. We have seen the disease reproduced in a stable where there was no possibility of contact with glandered matter, for the woodwork was either painted or removed, and the walls were re-plastered and the pavement altered; and although some months had elapsed since it was occupied by the glandered patient, and after these alterations were made, still the first horse that was put into it again took the disease and was destroyed. Dr. Burgess, who entertains similar views of the infectious nature of glanders, thus expresses himself on this subject:* "Glanders and farcy are essentially contagious diseases, whether developed in man or the quadrumana. They are, moreover, decidedly *infectious* as well as contagious in the latter, i. e. the contagious principle may be transmitted through the medium of the atmosphere, as well as by actual contact from one animal to another. I have known several instances in which there was no possibility of contact with glanderous matter, and yet the disease was developed in healthy horses. A gentleman of fortune in the west of Ireland had had his stud of horses infected with glanders. Every particle of woodwork in the stables, including stalls, rack, manger, &c., was taken down or replaced with new materials. The plastering on the walls was completely removed, and the pavement ripped up, and all was replaced with entirely new work; but the first horses that were again placed in these stables became infected, and they were ultimately razed to the ground. It would even appear that the contagious principle remains for a lengthened time, sometimes for years, in any stable or shed where glanders or farcy may happen to be developed." It is recorded that when the Duke of Marlborough took Lisle from the French, glanders broke out there among the horses with such virulence that the stables were obliged to be shut up, and remained so for thirty years, until another war broke out, when they were opened again, and the horses that were put into them became immediately glandered. That animals often escape the disease,

* Cazenave's Manual of Diseases of the Skin: art. Glanders. By T. H. Burgess, M.D. London, 1842.

although breathing a contaminated atmosphere, we freely admit, and have seen several remarkable instances of this kind; but that circumstance does not in the slightest degree affect the doctrine of infection. We have merely to refer to the general laws of contagion to render this argument against infection invalid. Six persons may visit a patient in typhus or scarlet fever, and one or two may contract the disease, whilst the others escape; yet no one would think of saying that typhus or scarlatina was not infectious, but that all these individuals were not equally susceptible of the disease. It would even appear that glanders has been communicated to man through the medium of the atmosphere, and Mr. Percival is labouring under a mistake when he supposes that man has taken the disease in no instance save through inoculation. (p. 225.) In the 'Lancet' for Feb. 11, 1832, the author will find a case of glanders transmitted from father to son without inoculation. In the Bull. de l'Acad. de Médecine, for Nov., 1841, he will find the case of a dresser who had the care of a glandered patient and contracted the disease, "not by inoculation, but in the same way that small-pox or scarlatina is contracted—by infection." And Dr. C. Williams relates a case of a girl sleeping over a stable where a glandered horse was kept, who became affected with a disease very analogous to glanders, although she did not come in contact with glanderous matter. (Med. Gazette, Dec., 1841.) Dr. R. Williams, in his work on 'Morbid Poisons,' relates another case. Also Tarozzi, an Italian writer, gives a graphic description of a pestilential disease which was developed in a stable where a glandered horse died. Out of thirty-five persons who visited that stable eleven were attacked with a malignant complaint, characterized, from its invasion to its termination, by fever and an eruption of boils and gangrenous pimples. These facts, however few, show us the possibility of contracting a malignant disease from the horse without contact, and teach us the necessity of precaution in going about glandered animals or their stables. That the majority of persons similarly situated escape infection is no argument against the danger of the disease.

3. The propagation of glanders by contact or inoculation is so fully established already, and especially as the author has nothing new on that subject, we shall not enter into it at present, but proceed at once to the next section of the work.

*Seat and nature of glanders.* That glanders and farcy are fundamentally the same disease, resulting from a common cause,—a specific poison—and differing from each other only in situation, is, we believe, now pretty generally admitted. That the aerial membrane, including that of the sinuses, is the *seat* of glanders proper, is also a proposition which the experience of veterinary pathologists has fully established. Dupuy, as we have already seen, was of opinion that glanders attacked the membrane of the frontal and other sinuses first, that of the nose next, and finally the lungs; whereas Rodet, the expounder of this doctrine, alleges that the schneiderian membrane is only affected secondarily, through extension of the disease from the lungs. Mr. Percival adopts the views of Dupuy on this point, in preference to those of Coleman, who considered the *septum nasi* to be the part first affected, and peculiarly susceptible of the disease. "Next to the sinuses of the head," says Mr. Percival, "we find the lungs taking on the disease; not by direct extension of the morbid action to them through the windpipe and its branches, but from the continual inhalation of the glanderous effluvia arising from the diseased surfaces in the nostrils and sinuses. The lungs, however, do not prove diseased in all cases of glanders." (p. 283.) The larynx is not infrequently the seat of glanders, but the author is of opinion that there is not the same susceptibility in the "membrane lining the windpipe as in other parts of it, or even in those divisions of it constituting or lining the pulmonary air-cells."

Mr. Percival enters, *con amore*, into the views of the pathology of glanders propounded by M. Leblanc, of Alfort, in an admirable monograph published by him in 1839. The author tells us that he is indebted to accident, a few months before writing his present article on glanders, "for a small work wherein, to his great satisfaction, he found notions entertained such as for many a year had been floating about in his own mind." It appears somewhat singular that M. Leblanc's important memoir should have escaped the notice of a writer of a text book on veterinary medicine for nearly five years, and then that he should be indebted to accident for its discovery. However, when he did find it out, he makes up for former negligence by adopting wholesale the doctrines of that excellent veterinarian. The principal novelty in M. Leblanc's views is—that glanders, like farcy, is a disease of the *lymphatic* system; that the pimples or tubercles observable in the incipient stages of glanders are nothing more than so many farcy-buds, which in time became pustules, burst, and end in turning to so many open, foul, and spreading ulcers,—in short, that the little eminences which Dupuy called "tubercles" are nothing more than so many farcy-buds, and that these consist of albuminous matter. M. Leblanc considers the pulmonary glanderous tubercle to be precisely the same morbid product as a farcy-bud, and not, as Dupuy supposed, identical with the tubercle of phthisis in man. Mr. Percival, with whom we perfectly agree, does not believe there is any analogy between the elementary lesion of glanders in the horse and that of phthisis in the human subject.

M. Leblanc thus explains the alteration which takes place in the lymphatic fluid during the progress of the disease. It first turns yellow and becomes coagulated within the canals of the lymphatics and the cavities of their glands, the tunics of the vessels thickening and turning opaque, exhibit red points upon their inner surface, and adhering in places to the coagula within, and in other places growing more or less softened, without, as yet, showing ulceration. In time all the thickened parts of the vessel partake of this softening, spreading from a single point upon its circumference, the coagulum within softening likewise, and the cellular tissue corresponding to the point of ulceration becoming tumefied, then hardening, and lastly softening. And now a little tumour exists, having its seat in part in the lymphatic vessel, in part in the cellular tissue, close upon the situation of the lymphatic valves, which accounts for the elevations, the lymphatic fluid in its incrassated or coagulated condition not being able to pass the valves. This explains the knotted aspect of the corded swellings in farcy. M. Leblanc explains his views of the nature of what has been called the "glanderous tubercle" in the following terms, with which Mr. Percival coincides:

"Examination of the mucous membrane of the nose of a glandered horse will show, in a certain stage, that it becomes *thickened.* And that this thickening, which is owing to an accumulation of fluids of a white or whitish-yellow colour, precedes the appearance of the tubercles, the same as tumefaction of the cellular membrane precedes the formation of farcy-buds. In this (thickened) condition the membrane assumes a shiny and more humid aspect than it has in health. Then, upon divers points of its surface, and notably upon the middle part of the nasal septum and within the doubling of the nostril, make their appearance little white or yellowish-white pimples (*élévures*), rather prominent at their centres, with borders insensibly declining to a level with the surrounding membrane. *These pimples or tubercles correspond to the course of the bundles of lymphatics, and very probably have their seat in those vessels.* 'At least,' continues Leblanc, 'I have been able to prove that little shreds (*masses élongées*), in composition absolutely like what is found within the lymphatics of a farcied limb, were inclosed within their canals, from which it was easy, with the points of the forceps (*d'un instrument*) to extract them: they proving adherent only in certain places, marked by some increase of redness. I have found the greatest analogy between these alterations and those which the lymphatic fluid commonly undergoes in farcy.'" (p. 288.)

We perfectly agree with Mr. Percival in his views of the specific nature of the *virus* of glanders, in fact, that no other animal poison will produce that disease but its own; but we differ from him when he says that the lymphatic system is not liable to derangement or inflammation from other causes. Septic matter, as Rayer observes (Cyclopædia of Surgery, art. Farcy), different from that either of farcy or glanders, may give rise in the quadrumana to inflammation of the lymphatic vessels and ganglions, accompanied by general phenomena of infection, and often terminating fatally; but nevertheless it is not glanders. We agree with the author in looking upon glanders as a *constitutional* and not a local disease. The constitutional disturbance, the eruption of the disease in other parts of the body in the form of farcy, the contaminated state of the blood, as shown by transfusion into another animal, and the inefficacy of topical remedies, are surely sufficient to prove that the disease is not local.

Farcy.—As the history of this disease is for the most part involved in that of glanders, we shall be brief in our remarks upon this part of Mr. Percival's work.

The disease called farcy consists of small tumours along the course of the lymphatic vessels, in the form of a knotted cord, which in time ripen into pustules, and terminate in ulceration. The disease most frequently attacks one or both of the hind legs. On viewing the limb from behind a fulness on the inside of the thigh may be seen, along the course of the femoral vein. Tracing the cord upwards from its place of origin, which is usually above the hock, the hand is carried into the groin, and there discovers a lobulated tumour, a swelling of the inguinal glands, which is called a bubo. The disease is sometimes developed in the course of a night, whilst on other occasions it appears in a more insidious manner. It may assume an acute, subacute, or chronic character. The solid buds gradually grow soft, from centre to circumference, and at length become *pustules* or little abscesses, which burst as soon as they are ripe. Instead of breaking, however, the farcy-bud sometimes hardens and becomes indolent, and remains so for a considerable time, but ultimately gives way, and, like the former, terminates in ulceration of a chancrous nature. Farcy seems to have a peculiar predilection for parts where the skin is thin and nearly free from hair. The inner aspect of the thighs and arms, the lips, nose, &c., are most commonly attacked. Mr. Percival seems to be at a loss to account for this result, although it seems clearly enough to be owing to the number of large vessels and lymphatics which traverse these regions. Coleman supposed the reason of the hind legs being most frequently affected was in consequence of their "distance from the central force of the circulation." Mr. Percival thinks it depends chiefly on the extra-work the hind legs have to perform in progression.

There is a variety of farcy called "button farcy," which does not select the course of the lymphatics especially for its seat, but is diffused in the form of numerous small tumours over the body, and as they remain small through all their stages, the author thinks they bear a resemblance to the *miliary* ulceration of glanders. They are also more intimately connected with the *cutis vera* than the other forms of farcy-bud. Farcy, especially when situated near the head, almost invariably terminates in acute glanders, and, on the other hand, M. Rayer observes, in the article already referred to, that the majority of horses which he saw labouring under acute farcy were also suffering from chronic glanders. This disease bears a striking resemblance to the diffused inflammation produced by dissection wounds, the lymphatics being the seat of both lesions. M. Hamont considers tubercular lepra of man to be identical with the farcy of the horse, and that the former disease is confined to the poorer classes of society, and never attacks the rich and well-fed, exactly as the latter is developed in an ill-fed and low breed of horses.

With reference to the *causes* of farcy, it may be stated that whatever produces glanders is also capable of producing that disease; for it cannot be otherwise if they are identical in their nature. For example, if an animal be inoculated with the virus of glanders, we cannot tell which disease will be developed; one is just as likely as the other, and we can only explain why glanders is produced instead of farcy, and *vice versâ*, by reference to the laws of predisposition.

Farcy has for its especial seat the skin, the *cutis vera;* that of glanders being the mucous membrane; and we entirely concur in the following remarks of Mr. Percival on this point:

"In strict pathology, glanders and farcy constitute one and the same disease of the lymphatic vessels and their glands; the disease originates in these vessels, and for a time confines itself to them; in the course of its progress, however, it extends into the contiguous tissues, affecting in one case the *cutis*, in the other the mucous lining of the air passages. No wonder, therefore, that the appearances of farcy should differ so much from those of glanders, and that the lesion of the one should be much more manageable than the other. Inflammation in the cutis is a different disease from that of a mucous membrane, productive of different phenomena, &c., hence the apparently wide differences between two diseases in their nature alike." (p. 318.)

Leblanc describes the farcy-bud to be the result of the coagulation of the lymphatic fluid accumulated in the vessels in an incrassated form, and obstructed by the valves, which latter, as Coleman first pointed out, are unsusceptible of the farcinous inflammation, and this will account for the peculiar plump spheroid shape of the farcy-bud, and of the pustule which

LIBRARY OF THE
Orleans Parish Medical Society succeeds it. The *skin* in the neighbourhood of the bud becomes indurated and thickened, sometimes to a remarkable degree. It becomes white, tough, and hard, cutting like so much white leather rather than skin, especially in the immediate vicinity of the buds, and several of the superficial buds which have already become pustular will be found imbedded in its thickened substance. When this indurated cartilaginous-like cutis is cut through, chains of farcy-buds and pustules are exposed, invested with cellular tissue, full of infiltration, of a jelly-like citron-coloured fluid. The disease sometimes extends deep between the muscles, forming abscesses, and in inveterate cases, according to Dupuy, even the bones of the limbs become involved.

With reference to the *treatment* of glanders Mr. Percival has nothing new to offer, and frankly admits that it is still the opprobium of his art. It is, in fact, an incurable disease, and, in this respect has for its analogue pulmonary consumption in man; either of these maladies once established may be palliated but not cured. Prophylactic measures alone are those from which we can expect any aid.

---

### ART. IV.

*Untersuchungen und Erfahrungen im Gebiete der Chirurgie.* Von Dr. FRIEDRICH PAULI.—*Leipzig,* 1844.

*Researches and Practical Observations in Surgery.* By Dr, F. PAULI.—*Leipsic,* 1844. 8vo.

